# Treadmill Exercise Preconditioning Attenuates Lung Damage Caused by Systemic Endotoxemia in Type 1 Diabetic Rats

**DOI:** 10.1155/2013/527090

**Published:** 2013-12-10

**Authors:** Ching-Hsia Hung, Jann-Inn Tzeng, Che-Ning Chang, Yu-Wen Chen, Chia-Ying Cho, Jhi-Joung Wang

**Affiliations:** ^1^Department of Physical Therapy, National Cheng Kung University, Tainan 701, Taiwan; ^2^Department of Food Sciences and Technology, Chia Nan University of Pharmacy and Sciences, Jen-Te, Tainan 717, Taiwan; ^3^Department of Anesthesiology, Chi-Mei Medical Center, Yong Kang, Tainan 710, Taiwan; ^4^Department of Physical Therapy, China Medical University, Taichung 404, Taiwan; ^5^Department of Medical Research, Chi-Mei Medical Centre, Tainan 710, Taiwan

## Abstract

Endotoxemia induces a series of inflammatory responses that may result in lung injury. However, heat shock protein72 (HSP72) has the potential to protect the lungs from damage. The objective of this study was to determine whether prior exercise conditioning could increase the expression of HSP72 in the lungs and attenuate lung damage in diabetic rats receiving lipopolysaccharide (LPS). Streptozotocin was used to induce diabetes in adult male Wistar rats. Rats were randomly assigned to sedentary or exercise groups. Rats in the exercise condition ran on a treadmill 5 days/week, 30–60 min/day, with an intensity of 1.0 mile/hour over a 3-week period. Rats received an intravenous infusion of LPS after 24 hrs from the last training session. Elevated lavage tumor necrosis factor-alpha (TNF-**α**) level in response to LPS was more marked in diabetic rats. HSP72 expression in lungs was significantly increased after exercise conditioning, but less pronounced in diabetic rats. After administration of LPS, exercised rats displayed higher survival rate as well as decreased lavage TNF-**α** level and lung edema in comparison to sedentary rats. Our findings suggest that exercise conditioning could attenuate the occurrence of inflammatory responses and lung damage, thereby reducing mortality rate in diabetic rats during endotoxemia.

## 1. Introduction

Pulmonary edema is a common complication of diabetes mellitus because of the increased capillary permeability [1]. Severely uncontrolled diabetic state may initiate pathologic events leading to the capillary leak of acute respiratory distress syndrome [2]. Due to the lower level and impaired binding activity of cell-surface receptors on monocytes [3], a poorly controlled diabetic state increases susceptibility to infections such as endotoxemia [4]. Additionally, infection can be more serious and difficult to eradicate in diabetic patients [5]. The highest incidence of acute respiratory distress syndrome occurred in endotoxemic patients and resulted in lung injury [6]. However, the severity of lung injury during systemic endotoxemiain type 1 diabetes remains unclear.

Endotoxemia is mainly caused by anendotoxin (lipopolysaccharide; LPS) from gram-negative bacteria [7]. Endotoxins induce a great amount of alveolar monocytes and macrophages to release tumor necrosis factor-alpha (TNF-*α*), which subsequently damages pulmonary vessels and increases lung vascular/epithelial permeability. This change allows more albumins to pass from the vessels to the alveolar space. Consequently, albumin content in the brochoalveolar lavage fluid increases significantly [8], contributing to lung edema and poor lung compliance. In unison, the alveolar distortion, atelectasis, and a thick alveolar-capillary membrane produce low efficiency of gas exchange, often resulting in increased mortality.

It is well known that regular exercise enhances cardiopulmonary function [9]. Prior research has demonstrated that heat shock protein72 (HSP72) can be detected in the lungs of exercise trained rats [10], and that its induction by heat can protect respiratory systems from systemic inflammation [11]. Moreover, HSP72 facilitates the action of an anti-inflammatory cytokine, interleukin (IL)-10, and alleviates TNF-*α* induced lung damage [12]. In nondiabetic rats, exercise training attenuated septic responses and abrogated pulmonary pathological change [13]. Swimming trained rats also showed a lower pulmonary edema index after LPS challenge [14]. In diabetic rats, exercise upregulated HSP72 expression in the heart and alleviated circulatory dysfunction after LPS injection [15]. Nevertheless, it remains unknown whether aerobic conditioning via treadmill exercise can attenuate pulmonary injury in diabetic rats receiving endotoxin. Hence, the purpose of this study is to reveal whether endotoxemia-induced lung injury is more marked in type 1 diabetes and to determine whether treadmill exercise conditioning could induce HSP72 overexpression in the lung, alleviate lung injury and proinflammatory cytokines overproduction in endotoxemia, and thus reduce the mortality rate.

## 2. Materials and Methods

### 2.1. Experimental Animals

Male Wistar rats (*n* = 144; 320 ± 20 g; aged 10 weeks) were purchased from the Animal Resource Center of the National Cheng Kung University in Taiwan. Rats were housed in groups of four at an ambient temperature of 24 ± 1°C. Pelleted rat chow and tap water were allowed *ad libitum*. All experimental procedures were conducted in compliance with the National Institutes of Health's *Guide for the Care and Use of Laboratory Animals*. Streptozotocin (STZ)-induced type 1 diabetic rats were prepared by intravenous injection with STZ (Sigma, St. Louis, MO, USA) (60 mg/kg) after a 72-hour fast. This method has been shown to irreversibly destroy pancreatic beta cells, as previously described [16]. After 1 week of STZ administration, rats with blood glucose levels higher than 300 mg/dL along with symptoms of polyuria were considered diabetic. Animals were separated into six groups (*n* = 24 in each group): (a) nondiabetic control rats receiving 0.9% normal saline administration (NS), (b) nondiabetic control rats receiving LPS administration (NL), (c) STZ-induced diabetic rats received normal saline administration (SS), (d) STZ-induced diabetic rats received LPS administration (SL), (e) STZ-induced diabetic rats subjected to exercise training before the injection of normal saline (SES), (f) STZ-induced diabetic rats subjected to exercise training before the injection of LPS (SEL).

### 2.2. Exercise Training Protocol

The exercise training protocol was performed according to a previously described method with some modification [17]. Specifically, 1 week after STZ injection, the trained rats ran gradually on a treadmill (treadmill exerciser T510, Diagnostic & Research Instruments, Singa) for 5 days/week with intensity of 1.0 mile/hr for 3 weeks at room temperature. Before the initiation of exercise training, the rats were acclimated to run 15 min at a time for 3 days. A minor electrical shock (1.0 mA) was used in the beginning to encourage rats to run forward. Subsequently, the animals ran without electrical stimulation. The duration of the exercise was progressively increased, with the rats running for 30 min/day during the first 2 weeks, and 60 min/day during the last week of training. The intensity of exercise was maintained throughout the training period at approximately 75–80% of maximal oxygen consumption [15, 18]. Same exercise training protocol is used in all training groups, close to 90% of the trained rats were able to finish the training program. Rats which could not achieve the intensity and duration of this training protocol were withdrawn from this experiment. We observed that the diabetic rats have lower body weight and lower endurance capacity. However, the withdrawal rate was indistinguishable between nondiabetic and diabetic group. At the 24th hr after the last training session, the rats received an intravenous injection of lipopolysaccharide (LPS, 15 mg/Kg) or saline. LPS (from *Escherichia coli* 0111:B4, Sigma, St. Louis, MO) was used as a fresh solution in phosphate buffered saline (pH 7.40) at a concentration of 10 mg/mL. After administration of LPS, the survival time of each rat was continuously monitored.

Different parts of the animals were used for three experiments (*n* = 8 for each part in each group): (I) determination ofsurvival time and survival rate in sedentary and exercised rats after receiving an injection of LPS; (II) determination of HSP72 expression in sedentary and exercised rats; and (III) determination of arterial blood gas, lung injury, and TNF-*α*, IL-6, and IL-10 levels in bronchoalveolar lavage at 240 min after normal saline or LPS injection.

### 2.3. Detection of Lung Vascular/Epithelial Permeability

The albumin content in bronchoalveolar lavage fluid was determined to assess the damage of endothelial cells within the lung capillaries. The rat's trachea was intubated and a bronchoalveolar lavage sample was collected by perfusing saline (15 mL) from the endotracheal tube at 240 min after the administration of LPS or saline. The lavage samples were centrifuged for 20 min (15000 rpm, 4°C) to remove cells. The supernatant of lavage was analyzed by a Bio-Rad protein assay system (Bio-Rad Hercules, CA) with bovine serum albumin as the standard. The albumin content in lavage was calculated by dividing albumin by the dried weight of the lung to evaluate pulmonary capillary/endothelial cell permeability.

### 2.4. Determination of Cytokine Concentration in Bronchoalveolar Lavage Fluid

The concentrations of TNF-*α*, IL-6, and IL-10 in lavage fluid were assayed using double-antibody sandwich ELISA (R&D Systems, Minneapolis, MN, USA) according to the manufacturer's instructions. The optical density of each well was determined by a microplate photometer (Multiskan EX, Thermo Fisher Scientific Inc, Waltham, MA, USA).

### 2.5. Measurement of Lung Edema

At 240 min after administration of LPS, both whole lungs were removed, wiped clean, and weighed as the wet weight. Dry weight was measured after the lungs were dried at 63°C for 48 hrs. In order to assess degree of lung edema, the wet/dry weight ratios of lung were calculated by dividing the wet weight by dry weight.

### 2.6. Histopathology of Lung

At 240 min after injection of LPS, the rats received 3 mL of air via tracheotomy with syringe. The systemic circulation was perfused with 250 mL of 0.9% saline (163.2 cm-H_2_O) while the pulmonary circulation was perfused with 50 mL of 0.9% saline (40.8 cm-H_2_O). Subsequently, the same amounts of 4% formalin were instilled into systemic and pulmonary circulation. Lung tissues were harvested and stored in 10% formalin for 3 days. Right upper lobes were embedded in paraffin blocks and serial sections (4 *μ*m in thickness) were stained with hematoxylin-eosin for histopathologic evaluation. The characteristics of lung damage include vascular congestion, hemorrhage, polymorphonuclear leukocytes (PMN) infiltration, and edematous changes of alveolar wall [19]. Each characteristic was scored (0: normal; 1: mild; 2: moderate; 3: severe) by a pathologist and overall lung injury was further calculated according to the sum of the scores.

### 2.7. Analysis of Arterial Blood Gas

In order to determine the arterial pH, arterial partial pressure of O_2_ (PaO_2_), CO_2_ (PaCO_2_), and O_2_ saturation (SO_2_) of rats, 0.4 mL of arterial blood was sampled from the femoral artery by a heparin-rinsed syringe at 240 min after administration of LPS. The sample was analyzed by a blood gas analyzer (Synthesis1725, Diamond Diagnostics Inc, Hollinton, MA, USA).

### 2.8. Western Blotting Analysis of HSP72

For purposes of quantifying HSP72 expression in the lungs, rats with or without exercise preconditioning were sacrificed 240 min after LPS injection. The rats' lungs were removed and stored at −70°C until analysis. Lung tissue was homogenized and denatured in a SDS sample buffer (0.5 M Tris-HCl (pH 6.8), 10% SDS, 0.1% bromphenol blue, 2-mercaptoethanol, and glycerol). Protein contents were assayed using the Bio-Rad kit (Bio-Rad, Hercules, CA, USA) and an ELISA reader (Multskan EX, Thermo, MA, USA) at 630 nm. After equal amounts of protein extract were loaded and separated by 10% SDS-polyacrylamide gel electrophoresis (SDS-PAGE), the proteins were transferred to polyvinylidene fluoride (PVDF) membranes (Millipore, Bedford, MA, USA). The membrane was incubated with a mouse monoclonal anti-HSP72 antibody (SPA 810; StressGen Biotechonologies, Victoria, BC, Canada). Immunodetection for HSP72 was performed using the enhanced chemiluminescence protocol using a Renaissance reagent (NEN Life Science Products, Boston, MA, USA). Mouse anti-actin monoclonal antibody was used as aninternal control. Quantification of blot band was performed using an optical scanner and ImageMaster TotalLab 1D Elite software (version 2.01; Amersham Pharmacia, Piscataway, NJ, USA).

### 2.9. Statistical Analysis

All values are presented as mean ± S.E.M. for each point. Statistical analysis was conducted using ananalysis of variance (ANOVA) for factorial experiments. The Wilcoxon rank-sum test was used for the analysis of survival time, while the Kaplan-Meier test was used for survival rates. Differences between groups were considered to be significant at values of *P* < 0.05.

## 3. Results

**Figure 1:**
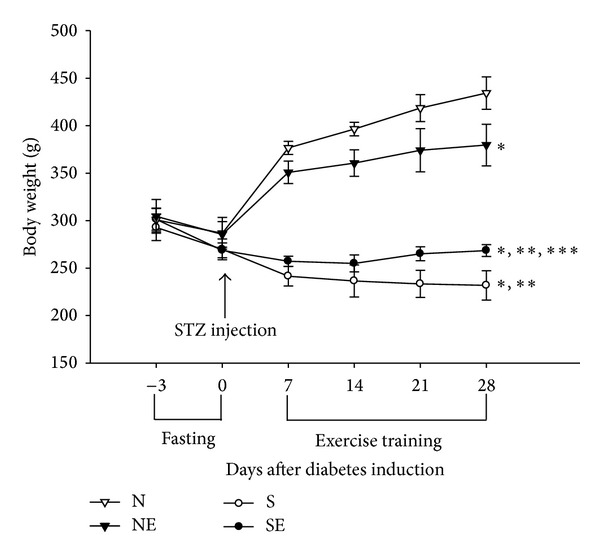
The changes of rats' body weight after STZ injection and 3-week exercise training. N: nondiabetic rats; NE: nondiabetic rats with exercise; S: STZ-induced diabetic rats; SE: STZ-induced diabetic rats with exercise (*n* = 8 per group). *P < 0.05, compared with the N group; **P < 0.05, compared with NE group, and ***P < 0.05, compared with the S group; (one-way repeat measurement ANOVA).

**Figure 2:**
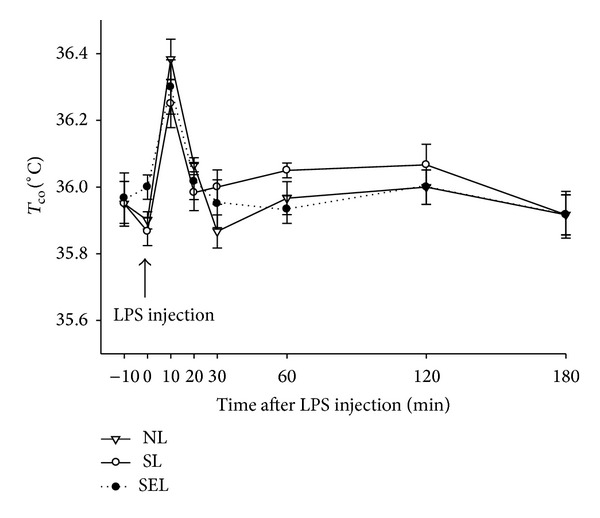
The colonic temperature (*T*_co⁡_) of the animals in response to LPS challenge. NL: nondiabetic rats receiving LPS administration; SL: STZ-induced diabetic rats injected with LPS; SEL: STZ-induced diabetic rats with exercise injected with LPS (*n* = 8 per group).

**Figure 3:**
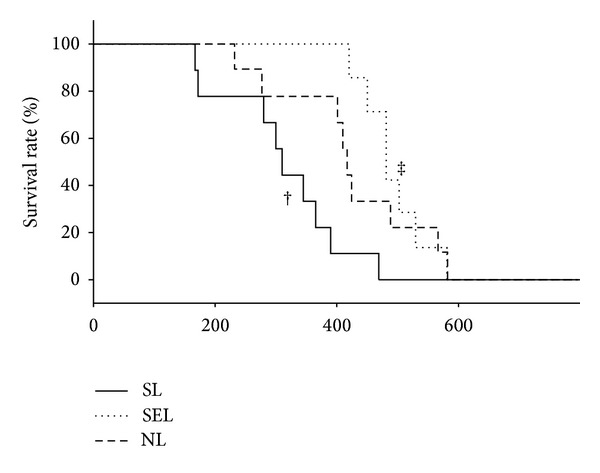
The survival rate of rats with or without exercise after LPS administration (15 mg/kg, i.v.). NL: nondiabetic rats receiving LPS administration; SL: STZ-induced diabetic rats injected with LPS; SEL: STZ-induced diabetic rats with exercise injected with LPS (*n* = 8 per group). ^†^ *P* < 0.05, compared with the NL group; ^‡^ *P* < 0.05, compared with the SL group (Kaplan-Meier test).

After induction of diabetes, the body weight of diabetic rats was lower than nondiabetic ones (Figure 1). Exercise training decreased the body weight of nondiabetic rats and increased the body weight of diabetic rats. In other words, exercise moves the body weight of rats toward a healthier status. After LPS administration, we found that the colonic temperature is slightly elevated within 10 mins and it returned to baseline level at 20 minutes (Figure 2). There are no significant difference within NL, SL, and SEL group. Therefore, LPS-induced fever is not altered by exercise training. The survival time of the SL group was markedly shorter than that of the NL group (316.4 ± 39.6 min versus 420.78 ± 38.99 min, resp.; *P* < 0.05). Additionally, survival time of the SEL group was significantly longer than that of the SL group (489.2 ± 23.5 min versus 316.4 ± 39.6 min, resp.; *P* < 0.05), suggesting that exercise preconditioning significantly improved survival rate of diabetic rats during endotoxemia (Figure 3). Presented in another way, by 420 min after administration of LPS, 100% of the SEL group was still alive in contrast to only 11.1% survival among the SL group.

**Figure 4:**
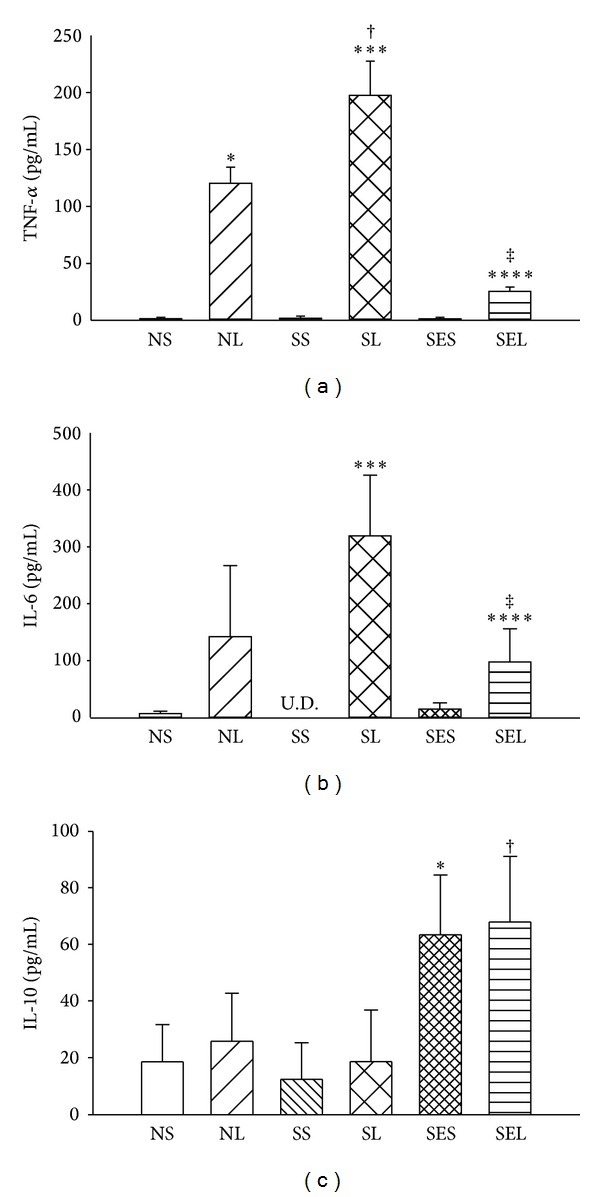
Levels of TNF-*α*, IL-6, and IL-10 in lavage fluid at 240 min after saline or LPS administration. NS: nondiabetic rats injected with saline; NL: nondiabetic rats injected with LPS; SS: STZ-induced diabetic rats injected with saline; SL: STZ-induced diabetic rats injected with LPS; SES: STZ-induced diabetic rats with exercise injected with saline; SEL: STZ-induced diabetic rats with exercise injected with LPS. Data are expressed as the mean ± SEM of eight rats per group. *P < 0.05, compared with the NS group; ^†^ *P* < 0.05, compared with the NL group; ***P < 0.05, compared with the SS group; ^‡^ *P* < 0.05, compared with the SL group; ***P < 0.05, compared with the SES group (one-way ANOVA).

Figure 4 shows that all groups treated with LPS displayed significantly raised TNF-*α* (120.34 ± 14.11 pg/mL in NL group and 197.43 ± 30.11 pg/mL in SL group) and IL-6 (142.11 ± 124.83 pg/mL in NL group and 319.11 ± 106.52 pg/mL in SL group) levels in lavage fluid at 240 min after treatment. However, lavage TNF-*α* level was statistically higher in the SL group as compared to the NL group (*P* < 0.05). Additionally, the increases of TNF-*α* and IL-6 were attenuated significantly by exercise preconditioning (25.5 ± 3.72 pg/mL in TNF-*α* and 97.52 ± 58.40 pg/mL in IL-6), while levels of IL-10 were actually higher in exercise-treated groups (68.50 ± 23.14 pg/mL).

**Figure 5:**
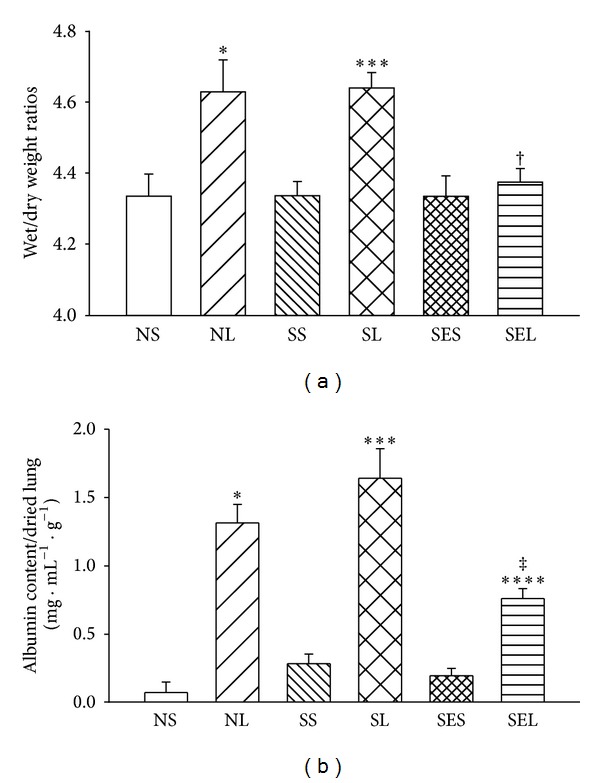
The lung wet/dry weight ratios and albumin content in lung lavage at 240 min after administration of saline or LPS. Albumin content in the brochoalveolar lavage fluid was calculated as [(mg of albumin)/(mL of lavage fluid)]/(g of dried lung weight). NS: nondiabetic rats injected with saline; NL: nondiabetic rats injected with LPS; SS: STZ-induced diabetic rats injected with saline; SL: STZ-induced diabetic rats injected with LPS; SES: STZ-induced diabetic rats with exercise injected with saline; SEL: STZ-induced diabetic rats with exercise injected with LPS. Data are expressed as the mean ± SEM of eight rats per group. *P < 0.05, compared with the NS group; ^†^ *P* < 0.05, compared with the NL group; ***P < 0.05, compared with the SS group; ^‡^ *P* < 0.05, compared with the SL group; ****P < 0.05, compared with the SES group (one-way ANOVA).

**Figure 6:**
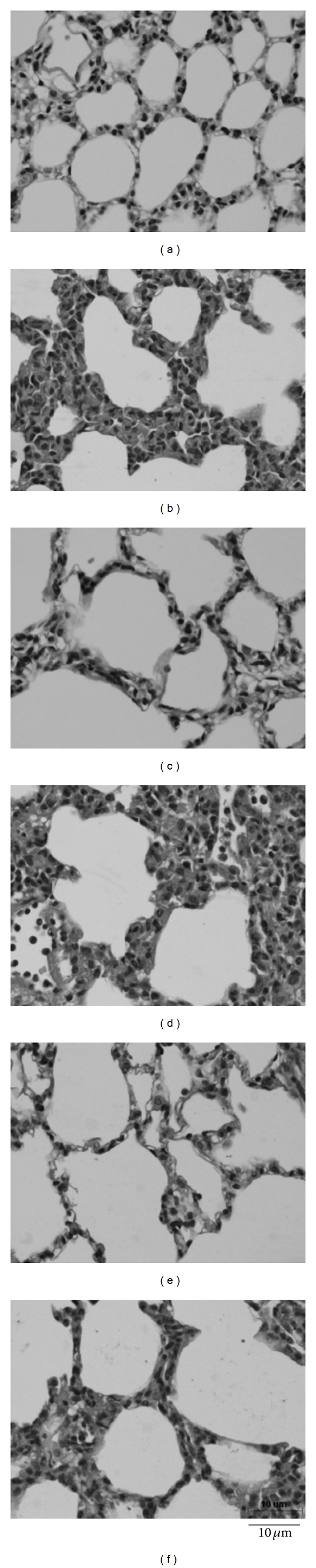
Histological examination of right upper lobe taken after 240 min of saline or LPS administration. (a) NS: nondiabetic rats injected with saline; (b) NL: nondiabetic rats injected with LPS; (c) SS: STZ-induced diabetic rats injected with saline; (d) SL: STZ-induced diabetic rats injected with LPS; (e) SES: STZ-induced diabetic rats with exercise injected with saline; (f) SEL: STZ-induced diabetic rats with exercise injected with LPS. The interstitial spaces of alveoli became wider after LPS administration, due to polymorphonuclear cell infiltration and edematous changes of alveolar walls (b), (d). Scale bar: 10 *μ*m.

**Figure 7:**
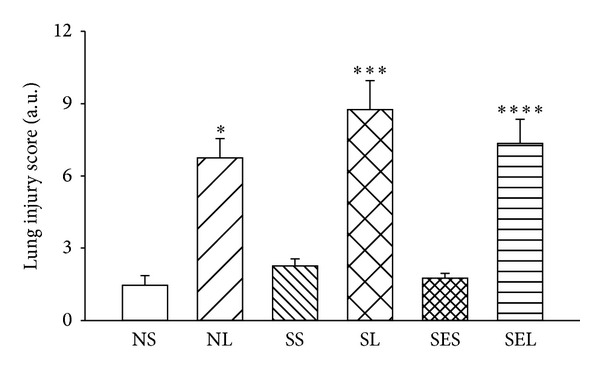
The lung injury score in different groups after LPS administration. Data are expressed as mean ± S.E.M. *P < 0.05, compared with the NS group; ***P < 0.05, compared with the SS group; ****P < 0.05, compared with the SES group (one-way ANOVA).

The lung wet/dry weight ratios and albumin content of the lungs increased significantly after administration of LPS (Figure 5). These parameters were not significantly different between the NL and SL groups, but the values were significantly decreased in rats with exercise preconditioning. Even though the albumin content in the SEL group was significantly lower than that in the SL group (0.76 ± 0.07 mg · mL^−1^ · g^−1^ versus 1.64 ± 0.22 mg · mL^−1^ · g^−1^, resp.; *P* < 0.05), the value of the SEL group was still significantly higher than that reported among the SES groups (0.76 ± 0.07 mg · mL^−1^ · g^−1^ versus 0.20 ± 0.05 mg · mL^−1^ · g^−1^, resp.; *P* < 0.05). Histopathologic examination of the lungs and quantitative analyses of histology were shown in Figures 6 and 7. The lung injury score was significantly increased in NL, SL, and SEL groups compared with that of NS groups. Although exercise preconditioning diminished these changes, the values of lung injury score are indistinguishable between SL and SEL groups. After administration of LPS, the values of PaCO_2_ decreased significantly, whereas the value of PaO_2_ and SaO_2_ increased significantly, indicating that hyperventilation had occurred. Nevertheless, values of PaCO_2_, PaO_2_, and SaO_2_ were indistinguishable between the SL and SEL groups (Table 1). Hence, the protective effect of exercise on blood gas changes is considered minor.

**Figure 8:**
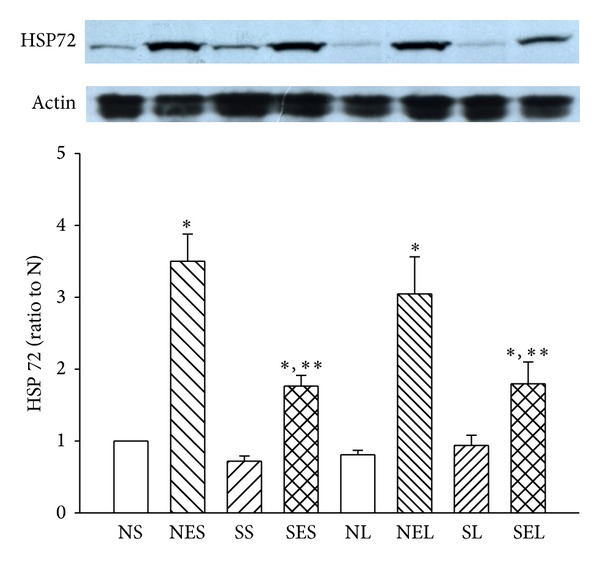
The expression of HSP72 in lungs in the rats with or without exercise after saline or LPS administration. NS: nondiabetic rats injected with saline; NES: nondiabetic rats with exercise injected with saline; SS: STZ-induced diabetic rats injected with saline; SES: STZ-induced diabetic rats with exercise injected with saline; NL: nondiabetic rats injected with LPS; NEL: nondiabetic rats with exercise injected with LPS; SL: STZ-induced diabetic rats injected with LPS; SEL: STZ-induced diabetic rats with exercise injected with LPS. Data are expressed as the mean ± SEM of eight rats per group. Protein levels are expressed as a ratio to the NS group. Below each column is a representative Western blot of HSP72 protein. Data are expressed as the mean ± SEM of eight rats per group. *P < 0.05, compared with the N group; **P < 0.05, compared with NE group (one-way ANOVA).

While levels of HSP72 expression in the lung were increased in rats with exercise as compared to those without exercise, the increase of expression was less pronounced in the diabetic groups (Figure 8). A negative correlation between lung HSP72 level and lavage TNF-*α* level was also demonstrated (*r* = −0.758, *P* < 0.05).

## 4. Discussion

In this study, we demonstrated that after LPS challenge, the TNF-*α* level in lung lavage fluid is higher in diabetic rats than that in nondiabetic ones. We also found overexpression of HSP72 in the lung and higher IL-10 level in lavage among exercised diabetic rats. Thus, our data suggest that preconditioning with treadmill exercise reduces lung edema, albumin content, TNF-*α* and IL-6 levels, and consequently, the mortality rate, after administration of LPS.

Lung injury is associated with elevated cytokines [20]. TNF-*α* is believed to play an important role in the pathogenesis of endotoxin-induced multiple organ failure [21]. Previous findings showed that LPS treatment resulted in higher lung TNF-*α* levels, as well as increased lung edema and pulmonary albumin [22]. However, HSP72 could facilitate the action of an anti-inflammatory cytokine, IL-10, thereby decreasing TNF-*α*-induced lung damage [12]. Several studies also demonstrated that HSP72 could actually inhibit the production of TNF-*α* [23] and TNF-*α*-induced inflammatory responses [24]. After LPS injection, we found that the colonic temperature is slightly elevated within 10 mins and it returned to baseline level at 20 minutes. Some evidence indicated that HSP72 is fundamental for survival at normal and raised temperatures. HSP72 also plays an important role in thermotolerance and cytoprotection against damage from stresses such as ischemia and cytokines [10, 17, 25]. In the present study, LPS-induced fever is not altered by exercise training. Nevertheless, exercise-induced HSP72 may provide cellular protection during the LPS-induced raised temperature period and lessen tissue inflammation and damage.

HSP72 is induced in multiple organs by heat stress or exercise [10, 26] and plays a critical role in the protection of cellular damage from such stressors [27]. More specifically, after exercise activates its generation, HSP72 interacts with denatured proteins causing refolding activity that intensifies the structure of normal proteins to prevent their degradation in response to a subsequent stress [25]. Additionally, HSP72 induced by heat was found to protect respiratory systems from systemic inflammation [28]. Previous study has demonstrated that the levels of other heat shock proteins, such as heat shock protein90 and heme oxygenase-1, are also changed in STZ-induced diabetic rats [29]. Induction of diabetes increased heme oxygenase-1 levels. Heat shock protein90 levels were increased in heart and decreased in liver of STZ-induced diabetic rats. However, exercise training significantly increased the expression of HSP72, but not heat shock protein90 and heme oxygenase-1, in heart, liver, and muscles of STZ-induced diabetic rats. Therefore, we focus on whether exercise training increases the expression of HSP72 in lung of STZ-induced diabetic rats in the present study.

LPS can induce proinflammatory cytokines, such as TNF-*α* or IL-6 [30]. Our data indicated that exercise training suppresses the LPS-induced inflammatory status. A prior study has reported that long-term exercise has an anti-inflammatory effect that is partially mediated by IL-6 [31]. After long-term exercise, IL-10 is stimulated by IL-6 in systemic circulation, which then causes a reduction in the TNF-*α* level. Furthermore, after 12 weeks of aerobic exercise, IL-6 level was found to be decreased while IL-10 level was increased in type 2 diabetic patients [32] and the patients with coronary heart disease [33].

In nondiabetic rats, recent studies documented that treadmill exercise attenuates septic responses and protects the lungs from damage [13]. Swimming trained rats also showed a lower pulmonary edema index after LPS challenge [14]. In the present study of diabetic rats, treadmill exercise increased the lung expression of HSP72, but this induction was less pronounced than in the nondiabetic controls. These data are consistent with prior reports indicating that induction of diabetes decreased HSP72 expression in heart, liver, and muscles [29]. Also, we found that exercised diabetic rats had lower inflammatory response during endotoxemia as well as a negative correlation between lavage TNF-*α* level and the HSP72 level in the lungs. A prior study has shown that activation of stress protein response caused intracellular expression of HSP72 in lung endothelial, epithelial, and macrophage cells and that this activation has immunomodulatory effects [34]. Hence, the attenuation of lavage cytokine levels during endotoxemia may be, in part, related to the higher expression of HSP72 in the lungs. Hence, we have demonstrated that LPS-induced pulmonary dysfunction is more marked in type I diabetic rats, but endurance training may upregulate lung HSP72 expression and counterbalance some of the negative effects of diabetes. However, since LPS injections affect multiple organs, the effect on survival time may not only be explained by the reduced severity of acute lung injury. In fact, our previous study has indicated that exercise could increase HSP72 expression in heart and NTS as well and attenuate cardiovascular dysfunction in diabetic rats during endotoxemia [15].

The present results showed that PaCO_2_ decreased and PaO_2_ increased significantly in diabetic rats during endotoxemia. LPS can induce many mediators, such as nitric oxide, reactive oxygen species, and TNF-*α*, all of which are associated with respiratory muscular dysfunction [35]. Furthermore, pulmonary morphology alterations can be involved in the changes of blood gas levels [36]. Our results are similar to those of Fakioglu et al. [37], who documented that hyperventilation occurred during endotoxemia, but inconsistent with findings from another study [38]. The controversial findings may be explained by different dosage of LPS injection in each study. In addition, some studies have demonstrated that alloxan-induced diabetes may be associated with a reduced risk of acute lung injury in response to intratracheal instillation of endotoxin [39]. The conflicting outcome in our study may result from the lung injury being induced by intravenous infusion of endotoxin and the use of a different animal model of diabetes.

Our results suggest that regular exercise could prevent the occurrence of a rapidly lethal infection or endotoxemia even among those with poorly-controlled diabetes. This finding may help the treatment of poorly controlled type 1 diabetes. Although exercise prolonged the survival time only by hours in this acute septic shock model, it could provide more opportunities for providing enough emergency treatments and critical care within these hours for the diabetic patients and reduce the higher mortality. Therefore, regular exercise is recommended for type 1 diabetic patient.

There are some limitations to this study. In present study, diabetic status is induced 1 week before the exercise protocol started. Therefore, exercise training is started from acute/young diabetic state. The tissue responses to exercise training may be different in chronic/prolonged diabetic state. A direct relationship between lung HSP72 content and related damage cannot be inferred since this study assessed the consequences separately. Such a question may be addressed by injecting HSP72 antisense or shRNA into the exercising rats to further clarify the effect of exercise-induced HSP72 expression on endotoxemia. Although the increase of TNF-*α* and IL-6 levels in exercised rats was alleviated after LPS challenge, a much broader panel of pro- and anti-inflammatory cytokines, chemokines, inflammatory mediators, and oxidative stress bio-markers also need to be measured in the lavage fluid as well as in other tissues and in systemic circulation. Since these data are derived from a rapidly lethal model of endotoxemia, the present results may not be extrapolated to less severe models.

In conclusion, LPS-induced lung inflammation and damage are more marked in type 1 diabetic rats. Exercise could alleviate lung damage and confer significant protection against the high mortality risk in diabetic rats during endotoxemia. This protective effect may be correlated with HSP72 overexpression in the lungs.

## Figures and Tables

**Table 1:** Effects of LPS administration (15 mg/kg, i.v.) on arterial blood gas in rats with or without exercise.

	pH	PaCO_2_ (mmHg)	PaO_2_ (mmHg)	SaO_2_ (%)
NS	7.38 ± 0.01	42.03 ± 1.08	106.95 ± 2.28	97.82 ± 0.17
NL	7.40 ± 0.02	30.66 ± 1.99*	122.09 ± 5.93*	98.77 ± 0.21*
SS	7.41 ± 0.01	41.96 ± 0.92	91.40 ± 2.64	96.93 ± 0.37
SL	7.43 ± 0.01	30.63 ± 1.58***	106.50 ± 3.55***	97.96 ± 0.23***
SES	7.39 ± 0.02	40.05 ± 1.35	89.92 ± 3.94	97.18 ± 0.21
SEL	7.41 ± 0.01	34.70 ± 0.92****	104.29 ± 4.83****	98.11 ± 0.25****

PaCO_2_: arterial carbon dioxide tension; PaO_2_: arterial oxygen pressure tension; SaO_2_: oxygen saturation. Data are expressed as means ± SEM of eight rats per group obtained 240 min after injection of LPS or saline. *P < 0.05, compared with the NS group; ***P < 0.05, compared with the SS group; ****P < 0.05, compared with the SES group (one-way ANOVA).

## Nomenclature

HSP72: Heat shock protein72
IL-6: Interleukin-6
IL-10: Interleukin-10
LPS: Lipopolysaccharide
NEL: Nondiabetic rats with exercise injected with LPS
NES: Nondiabetic rats with exercise injected with saline
NL: Nondiabetic control rats injected with LPS
NS: Nondiabetic control rats injected with saline
SEL: STZ-induced diabetic rats with exercise preconditioning injected with LPS
SES: STZ-induced diabetic rats with exercise preconditioning injected with saline
SL: STZ-induced diabetic rats injected with LPS
SS: STZ-induced diabetic rats injected with saline
STZ: Streptozotocin
TNF-*α*: Tumor necrosis factor-alpha.
